# Analysis of MRE11 and Mortality Among Adults With Muscle-Invasive Bladder Cancer Managed With Trimodality Therapy

**DOI:** 10.1001/jamanetworkopen.2022.42378

**Published:** 2022-11-16

**Authors:** Anthony M. Magliocco, Jennifer Moughan, David T. Miyamoto, Jeff Simko, William U. Shipley, Phillip J. Gray, Michael P. Hagan, Matthew Parliament, William J. Tester, Anthony L. Zietman, Susan McCarthy, Daryoush Saeed-Vafa, Yin Xiong, Taylor Ayral, Alan C. Hartford, Ashish Patel, Seth A. Rosenthal, Susan Chafe, Richard Greenberg, Michael A. Schwartz, Mark E. Augspurger, John A. Keech, Kathryn A. Winter, Felix Y. Feng, Jason A. Efstathiou

**Affiliations:** 1 H. Lee Moffitt Cancer Center, Tampa, Florida; 2 NRG Oncology Statistics and Data Management Center/ACR, Philadelphia, Pennsylvania; 3 Massachusetts General Hospital, Harvard Medical School, Boston, Massachusetts; 4 Department of Radiation Oncology, University of California, San Francisco; 5 Hunter Holmes McGuire Veterans Administration Medical Center, Richmond, Virginia; 6 Cross Cancer Institute, Edmonton, Alberta, Canada; 7 Einstein Medical Center Philadelphia, Philadelphia, Pennsylvania; 8 Dartmouth Hitchcock Medical Center, Lebanon, New Hampshire; 9 MD Anderson Cancer Center at Cooper, Camden, New Jersey; 10 Sutter Medical Group, Roseville, California; 11 Fox Chase Cancer Center, Philadelphia, Pennsylvania; 12 Mount Sinai Medical Center, Miami Beach, Florida; 13 Baptist MD Anderson Cancer Center, Jacksonville, Florida; 14 MultiCare Gig Harbor Medical Park, Gig Harbor, Washington

## Abstract

**Importance:**

Bladder-preserving trimodality therapy can be an effective alternative to radical cystectomy for treatment of muscle-invasive bladder cancer (MIBC), but biomarkers are needed to guide optimal patient selection. The DNA repair protein MRE11 is a candidate response biomarker that has not been validated in prospective cohorts using standardized measurement approaches.

**Objective:**

To evaluate MRE11 expression as a prognostic biomarker in MIBC patients receiving trimodality therapy using automated quantitative image analysis.

**Design, Setting, and Participants:**

This prognostic study analyzed patients with MIBC pooled from 6 prospective phase I/II, II, or III trials of trimodality therapy (Radiation Therapy Oncology Group [RTOG] 8802, 8903, 9506, 9706, 9906, and 0233) across 37 participating institutions in North America from 1988 to 2007. Eligible patients had nonmetastatic MIBC and were enrolled in 1 of the 6 trimodality therapy clinical trials. Analyses were completed August 2020.

**Exposures:**

Trimodality therapy with transurethral bladder tumor resection and cisplatin-based chemoradiation therapy.

**Main Outcomes and Measures:**

MRE11 expression and association with disease-specific (bladder cancer) mortality (DSM), defined as death from bladder cancer. Pretreatment tumor tissues were processed for immunofluorescence with anti-MRE11 antibody and analyzed using automated quantitative image analysis to calculate a normalized score for MRE11 based on nuclear-to-cytoplasmic (NC) signal ratio.

**Results:**

Of 465 patients from 6 trials, 168 patients had available tissue, of which 135 were analyzable for MRE11 expression (median age of 65 years [minimum-maximum, 34-90 years]; 111 [82.2%] men). Median (minimum-maximum) follow-up for alive patients was 5.0 (0.6-11.7) years. Median (Q1-Q3) MRE11 NC signal ratio was 2.41 (1.49-3.34). Patients with an MRE11 NC ratio above 1.49 (ie, above first quartile) had a significantly lower DSM (HR, 0.50; 95% CI, 0.26-0.93; *P* = .03). The 4-year DSM was 41.0% (95% CI, 23.2%-58.0%) for patients with an MRE11 NC signal ratio of 1.49 or lower vs 21.0% (95% CI, 13.4%-29.8%) for a ratio above 1.49. MRE11 NC signal ratio was not significantly associated with overall survival (HR, 0.84; 95% CI, 0.49-1.44).

**Conclusions and Relevance:**

Higher MRE11 NC signal ratios were associated with better DSM after trimodality therapy. Lower MRE11 NC signal ratios identified a poor prognosis subgroup that may benefit from intensification of therapy.

## Key Points

**Question:**

Is level of DNA repair protein MRE11 in bladder tumors associated with outcomes after bladder-preserving trimodality therapy in patients with muscle-invasive bladder cancer?

**Findings:**

In this prognostic study of patients pooled from 6 prospective clinical trials of trimodality therapy for muscle-invasive bladder cancer, higher normalized MRE11 protein expression was associated with significantly lower disease-specific mortality.

**Meaning:**

These results suggest that higher normalized levels of MRE11 are associated with better outcomes after trimodality therapy for muscle-invasive bladder cancer patients, while lower MRE11 may identify a poor prognosis subgroup that may benefit from intensification of therapy.

## Teaser

This prognostic study evaluates the association of higher normalized MRE11 protein expression with disease-specific mortality among patients undergoing trimodality therapy for muscle-invasive bladder cancer.

## Introduction

Although muscle-invasive bladder cancer (MIBC) is often treated with radical cystectomy, bladder-preserving trimodality therapy consisting of maximal transurethral resection of bladder tumor (TURBT) followed by chemoradiation can be an effective alternative.^1,2^ Multiple studies have demonstrated that trimodality therapy has similar long-term outcomes to radical cystectomy in selected patients^3,4,5,6,7^ while avoiding perioperative morbidity and preserving the quality-of-life of patients.^8^ Clinical criteria to select optimal candidates for trimodality therapy include T2 stage disease and absence of hydronephrosis or carcinoma in situ, but these are imperfect selection factors.^9^ There is an unmet need for prognostic and predictive biomarkers to precisely guide the optimal selection of patients appropriate for trimodality therapy.^10^

MRE11 is a DNA nuclease with an important role in coordinating response to DNA damage, including double-strand DNA breaks and stalled replication forks.^11^ MRE11 has been identified as a candidate predictive biomarker, where higher levels are associated with greater radiosensitivity, potentially due to a futile attempt to counteract intrinsic cellular deficiencies in DNA repair.^10^ A retrospective study using immunohistochemistry showed that high MRE11 levels (above the lowest quartile) were associated with better cancer-specific survival after radiotherapy for MIBC but not after radical cystectomy.^12^ These findings were supported in a separate retrospective study of patients treated with bladder preservation therapy, 83% of whom received concurrent chemotherapy.^13^ However, an analysis of 2 prospective United Kingdom bladder preservation therapy trials did not successfully validate MRE11 as a biomarker of radiation response.^14^ Of note, despite standard operating procedures for immunohistochemistry staining and interpretation, there was high variability and poor intercenter scoring agreement in MRE11 levels in this study, which may have limited the ability to discern significant associations between MRE11 and outcomes.^14^

Automated quantitative image analysis (AQUA) is a digital immunofluorescence technique that reproducibly quantifies protein expression and overcomes many methodological limitations of manual immunohistochemistry.^15^ In this study, a standardized CLIA-eligible AQUA assay was used to evaluate normalized MRE11 expression as a biomarker of response to trimodality therapy in MIBC patients enrolled in 6 prospective Radiation Therapy Oncology Group (RTOG) clinical trials (NRG/RTOG Nos. 8802, 9506, 9706, 9906, 0233, 8903).^4,16,17,18,19,20,21^

## Methods

### Clinical Cohorts

**Figure 1.**
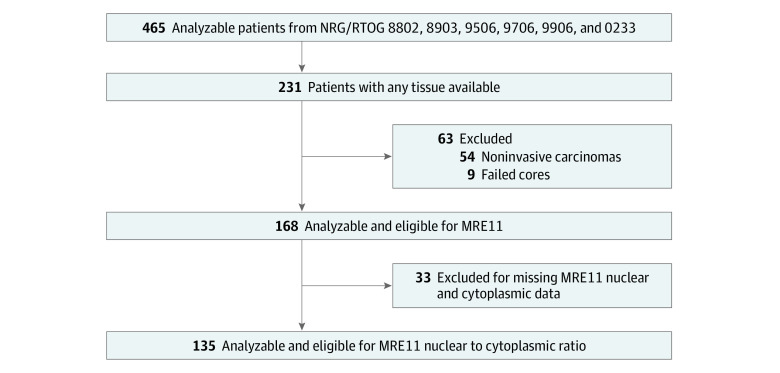
Participant Flow Diagram of Patients in this Study RTOG indicates Radiation Therapy Oncology Group.

Patients included in analysis had nonmetastatic MIBC, were enrolled in 1 of the 6 prospective RTOG clinical trials of trimodality therapy (NRG/RTOG Nos. 8802, 9506, 9706, 9906, 0233, 8903), and had tumor tissues analyzable for MRE11 expression and nuclear-to-cytoplasmic (NC) signal ratio (Figure 1). For each RTOG clinical trial, written informed consent was obtained, including for analysis of tumor tissues. Study approval was obtained from the institutional review board at Moffitt Cancer Center. This study follows most of the Transparent Reporting of a Multivariable Prediction Model for Individual Prognosis or Diagnosis (TRIPOD) reporting guidelines for prognostic studies, although it was not designed to build a full prediction model for individuals. Patient inclusion and exclusion criteria, characteristics, treatments received, and clinical outcomes are described in the publications reporting each trial,^16,17,18,19,20,21^ and are briefly summarized as follows:

#### RTOG 8802^16^

This phase II trial enrolled 90 analyzable patients with nonmetastatic MIBC at 21 institutions from 1988 to 1990, who received 2 cycles of neoadjuvant methotrexate/cisplatin/vinblastine (MCV) followed by once-daily radiation therapy to 39.6 gray (Gy) with concurrent cisplatin (70 mg/m^2^ every 3 weeks). Radiation therapy consisted of a small pelvic 4-field technique, encompassing the whole bladder, bladder tumor, prostate (in men), and adjacent pelvic lymph nodes.

#### RTOG 8903^17^

This phase III trial randomly assigned 123 analyzable patients with nonmetastatic MIBC from 1990 to 1993 at 37 institutions to 2 cycles of neoadjuvant MCV vs no MCV, followed by once-daily pelvic radiation therapy to 39.6 Gy and 2 cycles of concurrent cisplatin (100 mg/m^2^ every 3 weeks). Patients with a complete response on rebiopsy after 39.6 Gy of radiation therapy received consolidation therapy with 25.2 Gy of radiation therapy (total dose, 64.8 Gy) and 1 additional cycle of cisplatin.

#### RTOG 9506^18^

This phase I/II trial enrolled 34 analyzable patients with nonmetastatic MIBC from 1995 to 1997 at 11 institutions. After TURBT, patients received induction accelerated hypofractionated (3 Gy per fraction) twice-daily RT to the pelvis (24 Gy) with concurrent cisplatin (15 mg/m^2^ on days 1 to 3 and 15 to 17) and fluorouracil (400 mg/m^2^ on days 1 to 3 and 15 to 17). Patients with a complete response received twice-daily radiation therapy (2.5 Gy per fraction) to the whole bladder and bladder tumor (total dose, 44 Gy to bladder and tumor and 24 Gy to pelvic lymph nodes) with concurrent fluorouracil and cisplatin.

#### RTOG 9706^19^

This phase I/II study enrolled 47 analyzable patients with non-metastatic MIBC from 1997 to 1999 at 16 institutions. After TURBT, patients received induction twice-daily radiation therapy with 1.8 Gy to the pelvis in the morning and 1.6 Gy to the bladder tumor in the afternoon for 13 days (40.8 Gy to bladder tumor and 21.6 Gy to pelvis) with concurrent weekly cisplatin (20 mg/m^2^ first 2 days per week). Patients with a complete response on rebiopsy after 41.6 Gy of radiation therapy received twice-daily therapy (1.5 Gy per fraction) given to each site for 8 days (total dose, 45.6 Gy to pelvis and bladder and 64.8 Gy to bladder tumor) with weekly cisplatin.

#### RTOG 9906^20^

This phase I/II trial enrolled 78 analyzable patients with nonmetastatic MIBC from 1999 to 2002 at 21 institutions. After TURBT, patients underwent induction twice-daily accelerated radiation therapy over 13 days, including 1.6 Gy to the pelvis in the morning, 1.5 Gy to the bladder for the first 5 days (7.5 Gy), and then 1.5 Gy to the tumor for 8 days (12.0 Gy) in the afternoons (total dose, 20.8 Gy to pelvis, 28.3 Gy to whole bladder, and 40.3 Gy to bladder tumor) with concurrent weekly cisplatin (20 mg/m^2^ 2 days a week) and paclitaxel (50 mg/m^2^ per week). Patients with a complete response on rebiopsy after 40.3 Gy of radiation therapy received 1.5 Gy twice-daily pelvic radiation therapy to a dose of 24 Gy (total dose, 64.3 Gy to bladder tumor and 44.8 Gy to pelvis) with concurrent weekly cisplatin and paclitaxel. Patients received 4 cycles of adjuvant cisplatin (70 mg/m^2^) and gemcitabine (1000 mg/m^2^).

#### RTOG 0233^21^

This phase II randomized trial enrolled 93 analyzable patients with nonmetastatic MIBC from 2003 to 2007 at 24 institutions. After TURBT, patients received twice-daily radiation therapy, as in RTOG 9906, and were randomly assigned to either concurrent paclitaxel (50 mg/m^2^ per week) plus cisplatin (15 mg/m^2^ 3 days per week) or fluorouracil (400 mg/m^2^ 2 days per alternate week) plus cisplatin during induction and consolidation phases followed by adjuvant gemcitabine, paclitaxel, and cisplatin.

### Tissue Preparation and Immunofluorescence Assay for MRE11 Expression

A single 4 μm–thick section was cut from each pretreatment tumor tissue block, deparaffinized in xylene, rinsed in ethanol, and rehydrated. Heat-induced epitope retrieval was performed at 121 °C in citrate-based buffer (Dako) for 6 minutes in a decloaking chamber (Biocare Medical). Slides were stained using an autostainer (Dako). Endogenous peroxidase activity was quenched with peroxidase block for 5 minutes (Dako). Slides were washed with tris-buffered saline Tween-20 wash buffer (Dako) then incubated at room temperature for 30 minutes with signal stain protein block (Cell Signaling) containing a 1:1500 dilution of MRE11 rabbit monoclonal antibody clone EPR3471 (Epitomics) and pan-cytokeratin mouse monoclonal antibody (Dako). MRE11 signal was amplified and visualized using horseradish peroxidase-conjugated goat anti-rabbit antibody with cyanine 5 fluorophore-labeled tyramide signal amplification reagent (Akoya Biosciences). Pan-cytokeratin was visualized using a goat anti-mouse Alexa Fluor 555 secondary antibody (Invitrogen). Stained cells were mounted in mounting media with DAPI (4', 6-diamidine-2'-phenylindole dihydrochloride) (Vector Laboratories, Inc). All work was conducted in a clinical laboratory improvement amendments (CLIA)–grade laboratory by CLIA qualified personnel at the pathology tissue core facility at Moffitt Cancer Center.

### AQUA-Based Quantitation of MRE11 Expression

Stained and mounted slides were digitally scanned with an Aperio FL Scanscope, and the captured images were analyzed using AQUA software.^15^ Cellular compartments within image files were digitally mapped using DAPI signal to define the nuclear mask and cytokeratin signal to identify the epithelial area. Tumor mask was defined as cytokeratin signal plus nuclear signal. Tumor nuclear mask was defined as DAPI signal within cytokeratin positive areas. Using these masks, the MRE11 signal could then be measured within tumor nuclei and tumor cytoplasm. The AQUA score within each subcellular compartment was calculated by dividing the signal intensity by the total area of the specified compartment.^15,22,23^ A ratio of nuclear-to-cytoplasmic (NC) MRE11 AQUA score was calculated, and this was used as a normalized MRE11 score.

### Criteria for Complete Response and Patient Assessments

Clinical complete response was defined as no tumor palpable on bimanual examination under anesthesia, no tumor visible on cystoscopy, negative tumor site biopsy, and negative urine cytology per clinical protocol definition. Patients with preserved bladders underwent active surveillance including cystoscopy, tumor site biopsy, bimanual examination under anesthesia, and urine cytology every 3 months in year 1, cystoscopy and cytology every 3 to 4 months during year 2, every 6 months for 3 years, and then annually. Patients with non–muscle-invasive local failure were considered for intravesical therapy, and patients with muscle-invasive local failure underwent salvage radical cystectomy. All data were collected centrally at the NRG/RTOG Statistics and Data Management Center.

### End Points

All end points were measured from the date of study entry (phase II studies) or random assignment (phase III study) to the date of first documented event. For overall survival (OS) and disease-specific mortality (DSM), time was measured to date of death as a result of any cause or death from bladder cancer, respectively. For bladder-intact overall survival (BIOS), failure time was measured to the first occurrence of either cystectomy date or date of death due to any cause. Patients without the given end point event were censored at the time of last follow-up.

### Statistical Analysis

MRE11 NC ratios were analyzed as categorical variables using the lower quartile (Q1 or below vs above Q1) as a cut point. Statistical comparisons to assess potential associations between baseline characteristics and complete response with MRE11 NC ratio categories were carried out using the χ^2^ or Fisher exact test. In addition, comparisons between baseline characteristics for those patients with MRE11 NC ratio data and those without were also performed.

The Kaplan-Meier method^24^ was used to estimate OS and BIOS, and the log-rank test^25^ was used to compare MRE11 expression groups. Cumulative incidence^26^ was used to estimate DSM, and the Gray test^27^ was used to compare MRE11 expression groups. Death from any cause other than bladder cancer was considered a competing risk for DSM. Cox proportional hazards regression models^28^ were used to obtain hazard ratios (HRs) for OS and BIOS. Fine-Gray regression^29^ was used obtain HRs for DSM. For multivariable analyses, the following variables were assessed in the models along with MRE11 NC: age (below 70 years vs 70 years or older), sex (men vs women), race (White vs other), Eastern Cooperative Oncology Group (ECOG) performance status (0 vs 1), T stage (T2 vs T3/T4), and transurethral resection of bladder tumor (TURBT) (yes vs no or unknown). Race was categorized as White vs other because the sample sizes for other racial and ethnic groups were very small (with the exception of Asian patients). All analyses were conducted using SAS version 9.4 (SAS Institute). The threshold of significance was *P* < .05 in 2-sided tests. Analyses were completed in August 2020.

## Results

### Patient Characteristics

Of 465 patients with nonmetastatic MIBC enrolled in 6 prospective RTOG clinical trials of trimodality therapy, 168 patients had tumor tissues analyzable for MRE11 expression, of which 135 (80.4%) were analyzable for MRE11 NC ratio (Table 1). Of 135 analyzable patients, 111 (82.2%) were men with a median age of 65 years (minimum-maximum, 34-90 years). Most characteristics were not significantly different between groups, except that patients without analyzable tissue were more likely to be White (268 of 330 [81.2%] vs 87 of 135 [64.4%]; *P* < .001) and have T3 or T4 disease (148 of 330 [44.8%] vs 36 of 135 [26.7%]; *P* < .001).

**Table 1.** Characteristics of Analyzable vs Not Analyzable Patients for MRE11 NC Ratio

Patient or tumor characteristic	Analyzable, No. (%)		*P* value^a^
	No (n = 330)	Yes (n = 135)	
Age			
<70 y	207 (62.7)	92 (68.1)	.27
≥70 y	123 (37.3)	43 (31.9)	
ECOG performance status			
0	294 (89.1)	122 (90.4)	.68
1	36 (10.9)	13 (9.6)	
Sex			
Men	273 (82.7)	111 (82.2)	.90
Women	57 (17.3)	24 (17.8)	
Race			
White	268 (81.2)	87 (64.4)	<.001
Other^b^	62 (18.8)	48 (35.6)	
Histology			
Transitional	307 (93.0)	131 (97.0)	.09
Other	23 (7.0)	4 (3.0)	
T-stage			
T2	182 (55.2)	99 (73.3)	<.001
T3-T4	148 (44.8)	36 (26.7)	
Complete TURBT^c^			
Yes	284 (86.1)	117 (86.7)	.86
No/unknown	46 (13.9)	18 (13.3)	

Abbreviations: ECOG, Eastern Cooperative Oncology Group; NC, nuclear-to-cytoplasmic ratio; T-stage, tumor stage; TURBT, transurethral resection of bladder tumor.

^a^ *P* value from χ^2^ or Fisher exact test.

^b^ By race and ethnicity, other included: 42 (31.1%) Asian, 3 (2.2%) Black, 2 (1.5%) Hispanic, and 1 (0.7%) unknown.

^c^ Was a visibly complete TURBT performed?

### Normalization of MRE11 Expression by NC Signal Ratio

Tumor specimens from NRG/RTOG clinical trials span across multiple years and are susceptible to many possible sources of variation in absolute intensity of immunofluorescence signal, including sample age, ischemia, fixative type, fixation duration, time between cutting sections and staining, storage conditions, and other preanalytical tissue processing variables.^23,30^ In order to correct for variability and batch effects, MRE11 signal intensity was normalized by calculating an NC MRE11 signal ratio. The reasoning was that these preanalytical variables would likely lead to similar rates of MRE11 signal decay in both the nucleus and cytoplasm. Thus, the NC ratio should be resistant to variation across a wide range of conditions. When this approach was applied to MIBC samples, the MRE11 NC ratio was found to be not significantly different between older and newer cases (eFigure in the Supplement). Thus, normalization of MRE11 signal through the NC ratio minimizes batch effects resulting from antigen degradation due to preanalytical variables.

### MRE11 NC Signal Ratio and Clinical Outcomes

**Figure 2.**
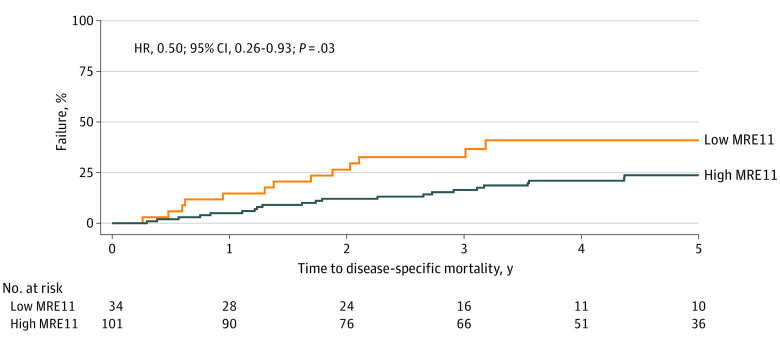
Cumulative Incidence of Disease-Specific Mortality (DSM) Low MRE11 nuclear-to-cytoplasmic (NC) ratio was defined as ≤1.49 (lower quartile), and high MRE11 NC ratio as >1.49.

The median MRE11 NC ratio across analyzable cases was 2.41 (first quartile, 1.49; third quartile, 3.34), and the mean was 2.52 (min-max, 0.69-6.03) (eFigure in the Supplement). The median (minimum-maximum) follow-up for alive patients was 5.0 (0.6-11.8) years. Remarkably, patients with a high MRE11 NC ratio above a binary cut point of 1.49 (defined by the first quartile threshold) had a significantly lower DSM compared with patients with a lower MRE11 NC ratio. The 4-year cumulative incidence of DSM for MRE11 NC ratio 1.49 or below was 41.0% (95% CI, 23.2%-58.0%) vs 21.0% (95% CI, 13.4%-29.8%) for ratios above 1.49 (HR, 0.50; 95% CI, 0.26-0.93; *P* = .03) (Table 2 and Figure 2). There were no significant differences by MRE11 NC ratio for OS, BIOS, or complete response (Table 2). In a multivariable regression model for DSM, after adjusting for other covariates, an MRE11 NC ratio above 1.49 was associated with a 58% decrease in DSM compared with a ratio of 1.49 or lower (HR, 0.42; 95% CI, 0.22-0.81; *P* = .009) (Table 3). Other significant covariates in the model included ECOG performance status (1 vs 0: HR, 2.69; 95% CI, 1.05-6.88; *P* = .04) and race (other vs White: HR, 0.39; 95% CI, 0.16-0.94; *P* = .04). The variables in the model were not correlated with each other. Together, these data show that higher normalized MRE11 NC ratio in bladder tumors was associated with lower DSM after bladder preserving trimodality therapy.

**Table 2.** Estimated Outcome and Complete Response by MRE11 NC Ratio (N = 135)

Measure	OS, % (95% CI)		DSM, % (95% CI)		Bladder-intact OS, % (95% CI)		Clinical complete response, No. (%) (n = 129)^a^	
	2 y	4 y	2 y	4 y	2 y	4 y	Yes	No
MRE11 NC								
≤1.49	70.6 (52.2-83.0)	51.4 (31.9-67.9)	26.5 (13.0-42.1)	41.0 (23.2-58.0)	64.7 (46.3-78.2)	49.4 (30.4-65.9)	20 (62.5)	12 (37.5)
>1.49	77.9 (68.4-84.9)	64.5 (53.9-73.2)	12.1 (6.6-19.3)	21.0 (13.4-29.8)	71.0 (61.0-78.9)	58.9 (48.3-68.0)	67 (69.1)	30 (30.9)
*P* value	.52		.03		.79		.49	
HR (95% CI)^b^	0.84 (0.49-1.44)		0.50 (0.26-0.93)		0.93 (0.55-1.58)		NA	

Abbreviations: DSM, disease-specific mortality; HR, hazard ratio; NA, not applicable; NC, nuclear-to-cytoplasmic ratio; OS, overall survival.

^a^ Six patients were not evaluable.

^b^ The reference level for HRs was an MRE11 NC signal ratio of ≤1.49.

**Table 3.** Multivariable Fine-Gray Regression Model of DSM^a^

Variables	Adjusted HR (95% CI)	*P* value^b^
MRE11 NC		
≤1.49	1 [Reference]	.009
>1.49	0.42 (0.22-0.81)	
Age		
<70 y	1 [Reference]	.25
≥70 y	1.51 (0.75-3.05)	
Sex		
Women	1 [Reference]	.06
Men	0.53 (0.28-1.03)	
Race		
White	1 [Reference]	.04
Other	0.39 (0.16-0.94)	
ECOG performance status		
0	1 [Reference]	.04
1	2.69 (1.05-6.88)	
T-stage		
T2	1 [Reference]	.48
T3-T4	0.78 (0.39-1.56)	
Complete TURBT		
No/unknown	1 [Reference]	.27
Yes	0.63 (0.28-1.42)	

Abbreviations: DSM, disease-specific mortality; ECOG, Eastern Cooperative Oncology Group; HR, hazard ratio; NC, nuclear-to-cytoplasmic ratio; T-stage, tumor stage; TURBT, transurethral resection of bladder tumor.

^a^ Results include 66 censored results, 42 events, and 72 competing events.

^b^ *P* value from the Fine-Gray regression model.

## Discussion

Bladder-preserving trimodality therapy is an increasingly accepted treatment option for MIBC^1,2^ that avoids the perioperative morbidity and decreased quality-of-life of radical cystectomy.^8^ Although clinicopathologic factors have been identified that correlate with outcomes after trimodality therapy,^9^ these can be insufficient as selection criteria, and reliable biomarkers are needed to select appropriate patients. To evaluate the DNA repair protein MRE11 as a prognostic biomarker, a standardized CLIA-eligible AQUA^15^ assay was used to quantitatively measure normalized MRE11 expression in pretreatment tumor tissues from patients enrolled in 6 prospective NRG/RTOG trimodality therapy clinical trials. A high MRE11 NC signal ratio above a binary cut point of 1.49 was found to be associated with a 4-year DSM of 21% compared with 41% in patients with lower MRE11 levels. The association between high MRE11 NC ratio and lower DSM remained significant in a multivariable regression model after adjusting for clinical covariates, including tumor stage. In the model, lower ECOG performance status and non-White race were also associated with lower DSM, although these findings require validation in additional cohorts.

Several candidate biomarkers for bladder preservation therapy have been identified involving DNA repair, signal transduction, proliferation, angiogenesis, and immune response.^10^ High MRE11 expression was previously associated with improved outcomes after radiotherapy for MIBC,^12,13^ but translation to clinical use has been challenging. Prior studies used proprietary research–grade immunohistochemistry methods and defined a binary cut point based on study population rather than absolute expression, thus limiting standardized implementation in a CLIA laboratory.^12,13^ The current study developed a standardized CLIA-eligible immunofluorescence assay to measure normalized MRE11 expression in formalin-fixed paraffin-embedded tissues through quantitation of the MRE11 NC signal ratio using AQUA.^15^ Unlike immunohistochemistry, immunofluorescence has high sensitivity and a large dynamic range, enabling precise measurement and simultaneous localization of multiple proteins. Normalization using an internal standard (nuclear compared with cytoplasmic signal) enabled establishment of an automated assay that overcomes bias from batch processing effects, preanalytical variables, and human error. This normalization strategy was based on the strong nuclear localization of MRE11 and likely background nature of cytoplasmic signal,^31,32^ although we cannot exclude the possibility that MRE11 NC ratio may have different biological implications than overall MRE11 expression. Using this CLIA-eligible assay, MRE11 NC ratio was found to be associated with better DSM after trimodality therapy in prospective clinical trial specimens.

The finding that higher levels of MRE11 in bladder tumors is associated with better outcomes after chemoradiation is consistent with prior studies.^12,13^ Although high expression of a DNA repair protein might be expected to associate with resistance to radiation-induced DNA damage, the observed association with greater sensitivity to DNA damage has also been noted in other contexts. In breast cancer, a lower recombination proficiency score resulting from higher expression of 4 DNA repair genes predicted a heightened sensitivity to DNA-damaging chemotherapy.^33^ One explanation is that higher levels of DNA repair genes may reflect a futile attempt of tumor cells to counteract intrinsic cellular deficiencies in DNA repair that make them more radiosensitive. There is also emerging data for a connection between MRE11 activity and cGAS-STING pathway activation, suggesting higher MRE11 levels may be associated with increased innate immune response.^34^ In contrast, lower levels of MRE11 identified a subgroup for whom trimodality therapy resulted in relatively high DSM. This poor prognosis subgroup may be more appropriately treated with radical cystectomy, or may benefit from treatment intensification through the addition of immune checkpoint inhibitors or targeted therapies to trimodality therapy.

### Limitations

This study has several limitations. Many cases in the NRG/RTOG pooled cohort did not have analyzable tissue, in part due to the age and prior usage of specimens from these historic trials. Further optimization of the AQUA method is needed to reduce attrition and reach commercial scalability. Nevertheless, the study was sufficiently powered to detect a significant prognostic value for MRE11 using available tissues. The study was not designed to determine whether MRE11 is a predictive biomarker, as suggested by prior studies.^12,13^ In addition, the contribution of other potential biomarkers of chemoradiation response, such as immune infiltration, were not examined.^35^ It will be important in future trimodality therapy studies to evaluate MRE11 in combination with other biomarkers, including other DNA repair gene alterations.

## Conclusions

In conclusion, normalized MRE11 expression in MIBC patients from NRG/RTOG bladder preservation trials is associated with outcome after trimodality therapy. Higher MRE11 NC ratio (ie, above 1.49 when measured using a standardized CLIA-eligible AQUA assay) was associated with lower DSM. Prospective validation in the SWOG/NRG 1806 trial (NCT03775265) and other studies could credential MRE11 and other biomarkers for the precise selection of patients for trimodality therapy, and for the identification of patients with poor prognoses who may benefit from treatment intensification.

## Notes

Supplement. **eFigure.** Standardization of MRE11 Expression by Nuclear/Cytoplasmic Ratio Reduces Batch Effects From Loss of Antigenicity Over Time
