# Supplementary material for: Analysis of MRE11 and Mortality Among Adults With Muscle-Invasive Bladder Cancer Managed With Trimodality Therapy
Source: JAMA Netw Open. 2022 Nov 16;5(11):e2242378. doi: 10.1001/jamanetworkopen.2022.42378 (PMC9669810; doi:10.1001/jamanetworkopen.2022.42378)
Supplement: Supplement. — eFigure. Standardization of MRE11 Expression by Nuclear/Cytoplasmic Ratio Reduces Batch Effects From Loss of Antigenicity Over Time [file jamanetwopen-e2242378-s001.pdf]

Supplemental Online Content

Magliocco AM, Moughan J, Miyamoto DT, et al. Analysis of MRE11 and mortality among adults with muscle-invasive bladder cancer managed with trimodality therapy. *JAMA Netw Open.* 2022;5(11):e2242378. doi:10.1001/jamanetworkopen.2022.42378

eFigure. Standardization of MRE11 Expression by Nuclear/Cytoplasmic Ratio Reduces Batch Effects From Loss of Antigenicity Over Time

This supplemental material has been provided by the authors to give readers additional information about their work.

© 2022 Magliocco AM et al. *JAMA Network Open.*

eFigure. Standardization of MRE11 Expression by Nuclear/Cytoplasmic Ratio Reduces Batch Effects From Loss of Antigenicity Over Time

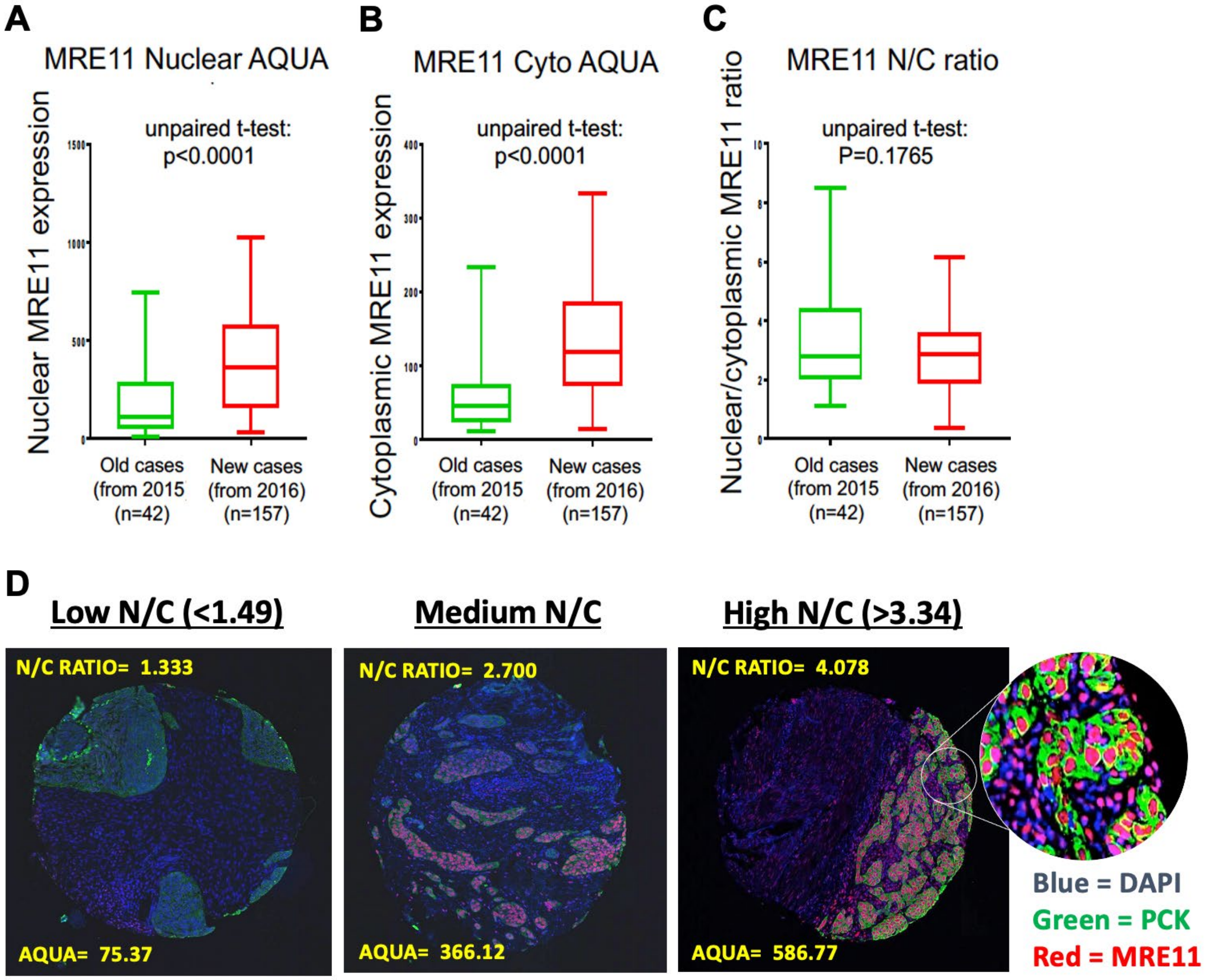

Standardization of MRE11 expression by Nuclear/Cytoplasmic Ratio reduces batch effects from loss of antigenicity over time. Box plots showing MRE11 signal in older cases compared to newer cases based on (**A**) nuclear MRE11 expression, (**B**) cytoplasmic MRE11 expression, and (**C**) nuclear/cytoplasmic MRE11 ratio. The top of the box plot represents the upper quartile (Q3), the bottom of the box plot represents the lower quartile (Q1), and the middle line in the boxplot is the median. The two whiskers of the plot extend out to the minimum and maximum value. (**D**) Representative images of muscle-invasive bladder cancer tissue microarray (TMA) cores following immunofluorescence staining for DNA (blue), pan-cytokeratin (green), and MRE11 (red). An analysis algorithm was used to calculate nuclear and cytoplasmic AQUA scores using pan-cytokeratin as the tumor mask. The AQUA score is defined as the average concentration of MRE11 pixel intensity within the tumor area of each TMA core. Panels from left to right show representative images of TMA cores with low, medium, and high MRE11 N/C ratios. Inset shows higher magnification view of a TMA core.

© 2022 Magliocco AM et al. *JAMA Network Open.*
